# Generation Mean Analysis Reveals the Predominant Gene Effects for Grain Iron and Zinc Contents in Pearl Millet

**DOI:** 10.3389/fpls.2021.693680

**Published:** 2022-01-28

**Authors:** Mahesh Pujar, Mahalingam Govindaraj, S. Gangaprasad, Anand Kanatti, T. H. Gowda, B. M. Dushyantha Kumar, K. M. Satish

**Affiliations:** ^1^International Crops Research Institute for the Semi-Arid Tropics, Patancheru, India; ^2^Department of Genetics and Plant Breeding, University of Agricultural and Horticultural Sciences, Shimoga, Shivamogga, India; ^3^Alliance of Bioversity International, International Center for Tropical Agriculture (CIAT), Cali, Colombia; ^4^Department of Biotechnology, University of Agricultural and Horticulatural Sciences, Shimoga, Shivamogga, India

**Keywords:** biofortification, iron (Fe), zinc (Zn), additive, dominant, gene interaction, epistasis, heritability

## Abstract

Pearl millet [*Pennisetum glaucum* (L.) R. Br.] is a climate-resilient dryland cereal that has been identified as a potential staple food crop that can contribute to alleviating micronutrient malnutrition, particularly with respect to grain iron (Fe) and zinc (Zn) contents, in Sub-Saharan Africa and India. In this regard, an understanding of the inheritance pattern of genes involved in Fe and Zn contents is vital for devising appropriate breeding methods to genetically enhance their levels in grains. In this study, we aimed to determine the genetic effects underlying such inheritance and their interactions based on the generation mean analyses. Four experimental crosses and their six generations (P_1_, P_2_, F_1_, BCP_1_, BCP_2_, and F_2_) were independently evaluated in a compact family block design in 2017 rainy and 2018 summer seasons. ANOVA revealed highly significant mean squares (*p* < 0.01) among different generations for grain Fe and Zn contents. Six-parameter generation mean analyses revealed a predominance of additive genetic effect and a significant (*p* < 0.05) additive × dominant interaction for the grain Fe content. The additive genetic effect for the grain Zn content was also highly significant (*p* < 0.01). However, interaction effects contributed minimally with respect to most of the crosses for the grain Zn content and hence we assume that a simple digenic inheritance pattern holds true for it. Furthermore, we established that narrow-sense heritability was high for the grain Fe content (>61.78%), whereas it was low to moderate for the grain Zn content (30.60–59.04%). The lack of superior parent heterosis coupled with non-significant inbreeding depression for Fe and Zn contents in grains further confirmed the predominance of an additive genetic effect. These findings will contribute to strategizing a comprehensive breeding method to exploit the available variability of grain Fe and Zn contents for the development of biofortified hybrids of pearl millet.

## Introduction

Pearl millet [*Pennisetum glaucum* (L.) R. Br.] is a climate-resilient dryland cereal crop predominantly grown for its grain and as fodder across the arid and semi-arid tropical regions of Africa (18 m ha) and Asia (10 m ha), particularly among poor farmers (FAOSTAT, 2020). With 8.6 million tons of grain produced per annum, India is the largest worldwide producer, with pearl millet being cropped on an area of approximately 7.0 million hectares (Directorate of Millet Development, 2020) and accounting for 20–60% (Dalberg, 2019) of the total cereal consumption among the major pearl millet growing states such as Maharashtra, Gujarat, and Rajasthan. Compared with other cereals, pearl millet is naturally a rich source of grain iron (Fe) and zinc (Zn) contents, which play a macro-role in human health. For example, Fe is a core component of hemoglobin that serves as an oxygen carrier in red blood cells, whereas both Fe and Zn play key roles in the functioning of numerous metabolic enzymes (Abbaspour et al., 2014).

Deficiencies in one or multiple essential nutrients can lead to micronutrient-associated malnutrition, also known as “hidden hunger” (UNICEF, 1990) with an excess of 2 billion people worldwide suffering from micronutrient deficiencies, primarily in developing countries such as Africa and India (World Health Organization [WHO], 2019). Within these populations, anemia has been found to be alarmingly high, particularly among pregnant women (40%) and children (42%) below 5 years of age (WHO)^1^. In India alone, 54% of pregnant women and 59% of children under the age of five are anemic, whereas 38% of children of the same age group are afflicted by stunted growth (NFHS, 2015–2016). Both anemia and stunting are, to a large extent, consequences of diet deficiency in Fe and Zn (Caulfield et al., 2006).

The biofortification breeding program of the International Crops Research Institute for the Semi-Arid Tropics (ICRISAT), supported by the Consortium of International Agricultural Research Centers (CGIAR) HarvestPlus, is continuously working toward the development of grain Fe- and Zn-biofortified pearl millet varieties and hybrids, and at present, the grains of commercially cultivated pearl millet varieties and hybrids have Fe and Zn contents of 42 and 32 mg kg^–1^, respectively (Rai et al., 2016). Genetic information on the inheritance of genes underlying grain Fe and Zn contents would further contribute to exploiting the potential inherent in the existing variability to attain the final breeding targets in the form of biofortified hybrids.

The genes underlying Fe and Zn assimilation being quantitative in nature are governed by a large number of genes that are strongly influenced by environmental conditions (Kumar et al., 2018). Frequently, these genes interact, thereby distorting Mendelian ratios, which in turn contributes to the development of novel phenotypes (Phillips, 1998). Estimates of gene action in crop improvement programs are assumed to have a direct bearing on the selection of breeding procedures to be followed. Earlier genetic studies based on hybrids and their parental lines have shown that both grain Fe and Zn contents are predominantly under additive genetic control (Velu et al., 2011; Govindaraj et al., 2013; Kanatti et al., 2014), and there is virtually no better parent heterosis (BPH). Furthermore, the *per se* performances of parental lines have been found to be significant and highly correlated with their general combining ability. However, although studies have expanded genetic models to estimate different genetic effects (Ribaut et al., 2012; Adebayo et al., 2014), most of these models, such as line × tester, diallel, and North Carolina design (NCD), are additive–dominance models or simply additive models, wherein epistatic or non-allelic interactions are rarely considered, thereby tending to lead to overestimations of gene action or underestimates of the contribution of gene interactions. It has, nevertheless, been established that inter-allelic interactions occur frequently and would have the effect of controlling the continuous expression of genes (Ribaut et al., 2012; Moharramnejad et al., 2015, 2016).

As an alternative, models based on generation mean analysis can take into account the effect or contribution of non-allelic interactions that have not been studied for these traits. The advantage of this type of analysis lies in the ability to provide information on the relative importance of the average effects of the genes (additive effects), dominance deviations, and effects due to non-allelic genetic interactions, which can assist in quantifying the genotypic value of individuals, and in turn, would contribute to determining the average generation genotypic value. In this regard, it is noteworthy that the magnitude and type of epistasis can have major repercussions with respect to both the reliability of predictions and the design of breeding strategies. Hence, in the present study, we sought to dissect the information regarding the classical inheritance gene interaction models using six basic generations evaluated in two contrasting seasons (rainy and summer) for grain Fe and Zn contents in pearl millet.

## Materials and Methods

### Plant Genetic Material

The genetic material used in the present study consisted of hybrid (F_1_) plants obtained from four crosses, along with their respective eight parents (Table 1). The selected parental inbred lines differed exclusively with respect to grain Fe and Zn contents (Table 2). Among the four crosses, crosses I, II, and IV were associated with the B × B group, whereas only cross-III was from the R × R group. This is a random contrast parent’s selection and crosses made specific to B and R groups for better understanding and possible use in hybrid-parent breeding. These inbreds, which were developed at International Crops Research Institute for the Semi-Arid Tropics (ICRISAT), Patancheru, Telangana, India, also differed in terms of performance for important agronomic traits, including flowering time, plant height, thousand-grain weight, and panicle size.

**TABLE 1:** Parentage of pearl millet inbred lines (P_1_ and P_2_) involved for the study of generation mean analysis.

Crosses	P_1_ (male)	Male pedigree	Crossed	P_2_ (female)	Female pedigree
Cross-I	ICMB 100489	EEDBC S1-425-2-1-2-4-B-2-3-B-B	×	ICMB 100478	[[(ICMR 312 S1-1-5-2-B × HHVBC)-10-2-1-2-3 × EEBC 407)-7-2-1x{EEBC S1-407-1-B-B-B-B-B-1-B-1-B-5-1x 3981-3989 G1}-2-1-1]-11-3
Cross-II	ICMB 100242	((SRC II C3 S1-19-3-2 × HHVBC)-27-1-3-3-3-3-2 × {[(843B × ICTP 8202-161-5)-20-3-B-B-3 × B-bulk]-2-B-1-2-2-B-B-B-11-1 × B-bulk (3981-4011/S06 G1)}-3-2-4-4)-35-2-5	×	ICMB 100245	(ICMB 98222xMRC HS-130-2-2-1-B-B-3-B-B-B-1-3-1)-11-2
Cross-III	ICMR 100775	[MRC HS-130-2-2-1-B-B-3-B-B-B-1-3-1 × {[(((IP 12322-1-2) × B-Lines)-B-14) × (MRC S1-156-2-1-B)]-B-1-3-3-B} × {GB 8735-S1-15-3-1-1-3-4-2-2-2-1}-B-11-5-1-1-1]-20-1-B	×	ICMR 100015	{((MC 94 S1-34-1-B × HHVBC)-16-2-1) × (IP 19626-4-2-3)]-B-28-3-1-2-2} × {MRC HS 225-3-5-2-B-B-B-B}-B-4-2-1
Cross-IV	ICMB 100474	[(MC 94 S1-34-1-B × HHVBC)-10-4-3-2 -2-B-B-2 × (ICMR 312 S1-1-5-3-B × HHVBC)-7-1-1-1-B-B-B]-21-B-1-4-1-2-1	×	ICMB 100410	(ICMB 04888 × ICMB 02333)-3-1-2-3

**TABLE 2:** Estimates of mean and range for grain iron (Fe) and zinc (Zn) contents among different generations within each cross evaluated in 2017 rainy (E_1_) and 2018 summer (E_2_).

Crosses	Generation	Iron (Fe)				Zinc (Zn)			
		E_1_		E_2_		E_1_		E_2_	
		Mean	Range	Mean	Range	Mean	Range	Mean	Range
Cross-I	P_1_	41	–	52	–	23	–	32	–
	P_2_	79	–	80	–	44	–	50	–
	F_1_	53	–	69	–	34	–	42	–
	F_2_	55	28–92	73	48–123	36	17–74	42	28–69
	BCP_1_	47	31–72	64	42–79	31	17–58	38	26–49
	BCP_2_	63	41–87	74	50–95	41	21–62	44	29–59
Cross-II	P_1_	49	–	48	–	31	–	35	–
	P_2_	60	–	75	–	41	–	43	–
	F_1_	50	–	59	–	35	–	38	–
	F_2_	51	28–102	63	35–119	36	21–83	40	23–85
	BCP_1_	48	30–69	58	33–79	34	20–77	37	22–59
	BCP_2_	58	38–99	67	40–115	37	25–63	41	25–62
Cross-III	P_1_	105	–	106	–	52	–	57	–
	P_2_	64	–	70	–	36	–	45	–
	F_1_	59	–	71	–	37	–	44	–
	F_2_	72	27–128	83	32–142	38	20–82	47	25–79
	BCP_1_	81	46–128	87	56–122	42	23–82	49	30–75
	BCP_2_	64	40–89	74	45–97	36	21–54	43	25–67
Cross-IV	P_1_	57	–	55	–	35	–	36	–
	P_2_	90	–	103	–	43	–	46	–
	F_1_	63	–	82	–	36	–	42	–
	F_2_	63	40–92	72	42–114	36	20–68	39	24–69
	BCP_1_	58	30–80	67	45–88	35	22–61	37	22–54
	BCP_2_	68	54–100	74	50–95	35	22–52	40	26–55

*E_1_, 2017 rainy; E_2_, 2018 summer; P_1_, Female; P_2_, Male; BCP_1_, F_1_ × P_1_; BCP_2_, F_1_ × P_2_; F_2_, selfed F_1_.*

### Development of F_2_, BCP_1_, and BCP_2_ Generations

Four single-cross hybrids (F_1_) were developed using selected parents (P_1_ and P_2_) with contrasting grain Fe and Zn characteristics. The crossing program was performed in the 2017 summer to develop segregating generations, i.e., backcross and second filial (F_2_) generations. In each cross combination, a BCP_1_ generation was developed by crossing F_1_ individuals with the respective parent P_1_ (female parent). Similarly, BCP_2_ generation progeny was obtained by crossing F_1_ individuals with parent P_2_ (male parent). Finally, the F_2_ populations for each cross were developed by selfing F_1_ plants.

### Crossing Program

In the backcross block, the panicles of male and female parental line plants were bagged using 30 cm × 10 cm parchment paper bags at the boot leaf stage to avoid contamination by foreign pollen. When developing BCP_1_ and BCP_2_ generations, both P_1_ and P_2_ plants were used as pollen sources, and F_1_ plants were used as a female, i.e., pollen collected from P_1_ plants was used to pollinate F_1_ plants to develop BCP_1_ generation progeny, whereas pollen from P_2_ plants was used to pollinate F_1_ plants to develop the BCP_2_ generation. The bagging of male and female line plants was performed daily. At the full bloom stage (detected by the observation of protruding white feathery stigma on the female parent), pollen (bulk pollen) from the male parent (P_1_ and P_2_) was collected in a parchment paper bag and dusted on the female lines (F_1_) by gentle thorough tapping in the morning hours between 08:00 a.m. and 11:30 a.m. Soon after the pollination, the crossed panicles were covered with parchment paper bags to avoid foreign pollen contamination, appropriately labeled, harvested at maturity, sun-dried for more than 15 days, and threshed to collect hybrid seeds. Prior to threshing, we removed one-third of the top and bottom of the panicles to ensure the high quality of crossed seeds and to minimize the likelihood of collecting any selfed seeds.

### Field Evaluation

The six generations, (P_1_, P_2_, F_1_, BCP_1_, BCP_2_, and F_2_) for each cross were evaluated in a compact family block design (CFBD) with three replications at ICRISAT, Patancheru, in two contrasting seasons, namely the 2017 rainy (E_1_) and the 2018 summer (E_2_). The rainfall, temperature, and relative humidity during field evaluation in E_1_ and E_2_ are presented in Supplementary Tables 1, 2, respectively. The CFBD consisted of a block comprising two rows each of P_1_, P_2_, and F_1_; six rows each of BCP_1_ and BCP_2_; and eight rows of F_2_, thereby ensuring a sufficient number of plant samples per generation. Sowing was carried out using a tractor-mounted four-cone planter (7100 US model, John Deer, Moline, IL, United States) with seeds planted in 4-m-long rows spaced at 75 and 60 cm in E_1_ and E_2_, respectively. All recommended agronomic practices were followed for good and healthy crop growth. Observations were recorded only for grain Fe and Zn contents in 15 plants of the P_1_, P_2_, and F_1_ generations, whereas we selected 120 BCP_1_ and BCP_2_ plants. Among the F_2_s, 300 plants were selected together across three plots. Soil samples were collected from the experimental fields from depths of between 0 and 30 cm and bulked to prepare single composite samples. Soil micronutrient analysis was performed using the diethylenetriaminepentaacetic acid extractable method (Lindsay and Norvell, 1978) at the Charles Renard Analytical Laboratory, ICRISAT, Patancheru. The mean values obtained for the soil Fe and Zn contents at ICRISAT, Patancheru in the E_1_ and E_2_ were 3.8 and 2.0 mg kg^–1^ and 5.0 and 1.6 mg kg^–1^, respectively. The soil contents of these two elements were accordingly deemed to be within a range sufficient for normal plant requirements (2.6 to 4.5 mg kg^–1^ for Fe; 0.6 to 1.0 mg kg^–1^ for Zn) (Tisdale et al., 1993; Sahrawat and Wani, 2013).

### Grain Sampling and Micronutrient Analysis

Open-pollinated grains were sampled and used to obtain estimates of grain Fe and Zn contents, which are expressed as mg kg^–1^. At the time of harvesting, 5–10 representative main panicles from plants growing in each plot were harvested at physiological maturity (80–90 days after planting) for P_1_, P_2_, and F_1_ generation plants, whereas, for the BCP_1_, BCP_2_, and F_2_ generations, we separately harvested single plant panicles (tagged plants). The harvested panicles were placed in a separate cloth bag to avoid soil contamination and dried in the sun. The dried panicles were threshed manually, and samples of approximately 20 g of grain were collected for grain Fe and Zn content analyses. Grains were cleaned of the glumes, panicle chaff, and debris, and thereafter transferred to non-metal fold envelopes and stored at a cold temperature (12°C), with care being taken at each step to avoid contaminating the grains with dust, following Stangoulis and Sison (2008).

Grain Fe and Zn contents were analyzed using an X-Supreme 8000 energy-dispersive X-ray fluorescence spectrometry device (ED-XRF: Oxford Instruments, Abingdon, United Kingdom), installed at the HarvestPlus Lab, ICRISAT, Patancheru. The XRF method used for pearl millet has been calibrated and validated in Flinders University, Adelaide, Australia (Paltridge et al., 2012) and ICRISAT (Govindaraj et al., 2016).

### Statistical Analysis

#### Generation Mean Analysis

The data obtained were subjected to ANOVA using a compact family block design, as described by Panse and Sukhatme (1985). The four scaling tests (A, B, C, and D) were determined according to the method suggested by Mather (1949). These different scales are computed simply by simple linear combination as given below:


$$\mathrm{ScaleA}=2\,\bar{{\text{BCP}}_{1}}-{\bar{\text{P}}}_{1}-{\bar{\text{F}}}_{1}=0$$

$$\mathrm{ScaleB}=2\,\bar{{\text{BCP}}_{2}}-{\bar{\text{P}}}_{2}-{\bar{\text{F}}}_{1}=0$$

$$\mathrm{ScaleC}=4\,{\bar{\text{F}}}_{2}-2\,{\bar{\text{F}}}_{1}-{\bar{\text{P}}}_{1}-{\bar{\text{P}}}_{2}=0$$

$$\mathrm{ScaleD}=2\,{\bar{\text{F}}}_{2}-{\bar{\text{BCP}}}_{1}-{\bar{\text{BCP}}}_{2}=0$$

Where ${\bar{\text{P}}}_{1}$, ${\bar{\text{P}}}_{2}$, ${\bar{\text{F}}}_{1}$, ${\bar{\text{F}}}_{2}$, ${\bar{\text{BCP}}}_{1}$, and ${\bar{\text{BCP}}}_{2}$ are means of different generations, respectively. The variances of the quantities A, B, C, and D were calculated from respective variances of different generations as given below:


$$\text{VA}=4\,\text{V}\,\left({\bar{\text{BCP}}}_{1}\right)+\text{V}\,\left({\bar{\text{P}}}_{1}\right)+\text{V}\,\left({\bar{\text{F}}}_{1}\right)=0$$

$$\text{VB}=4\,\text{V}\,\left({\bar{\text{BCP}}}_{2}\right)+\text{V}\,\left({\bar{\text{P}}}_{2}\right)+\text{V}\,\left({\bar{\text{F}}}_{1}\right)=0$$

$$\text{VC}=16\,\text{V}\,\left({\bar{\text{F}}}_{2}\right)+4\,V\,\left({\bar{\text{F}}}_{1}\right)+\text{V}\,\left({\bar{\text{P}}}_{1}\right)+V\,\left({\bar{\text{P}}}_{2}\right)=0$$

$$\text{VD}=4\,\text{V}\,\left({\bar{\text{F}}}_{2}\right)+\text{V}\,\left({\bar{\text{BCP}}}_{1}\right)+\text{V}\,\left({\bar{\text{BCP}}}_{2}\right)=0$$

Where, VA, VB, VC, and VD are the variances of respective scales A, B, C, and D; $\text{V}\,{\bar{\text{P}}}_{1}$, $\text{V}\,{\bar{\text{P}}}_{2}$, $\text{V}\,{\bar{\text{F}}}_{1}$, $\text{V}\,{\bar{\text{F}}}_{2}$, $\text{V}\,{\bar{\text{BCP}}}_{1}$, and $\text{V}\,{\bar{\text{BCP}}}_{2}$ are the variances of P_1_, P_2_, F_1_, F_2_, BCP_1_, and BCP_2_ generations, respectively. SEs for A, B, C, and D scales were calculated by estimating the square root of the respective variances. The test of deviation from the hypothetical value of zero was tested using the *t*-test. The calculated *t*-values were compared with “*t*” table values at 5 and 1% level of significance at their respective degrees of freedom. In each test, the degrees of freedom was taken as the sum of the degrees of freedom of various generations involved in that scaling test, and the degrees of freedom for any generation was calculated as a total number of observations minus the number of replications.

After computing scaling tests, if any one of them was found significant then the genetic effects were estimated by fitting the data into six-parameter models of the generation mean analysis, as suggested by Hayman (1958) to estimate the genetic parameters *viz.*, mean (*m*), additive gene effects (*d*), dominance gene effects (*h*), and three types of non-allelic gene interactions *viz*., additive × additive (*i*), additive × dominance (*j*), and dominance × dominance (*l*).


$$m=\text{Mean}={\bar{\text{F}}}_{2}$$

$$d=\mathrm{Additive}\mathrm{effect}={\bar{\text{BCP}}}_{1}-{\bar{\text{BCP}}}_{2}$$

$$h=\mathrm{Dominance}\mathrm{effect}={\bar{\text{F}}}_{1}-4{\bar{\text{F}}}_{2}-\left(1/2\right){\bar{\text{P}}}_{1}-\left(1/2\right){\bar{\text{P}}}_{2}+2{\bar{\text{BCP}}}_{1}+2{\bar{\text{BCP}}}_{2}$$

$$i=\text{Additive}\times \mathrm{Additive}\mathrm{effect}=2{\bar{\text{BCP}}}_{1}+2{\bar{\text{BCP}}}_{2}-4{\bar{\text{F}}}_{2}$$

$$j=\text{Additive}\times \mathrm{Dominance}\mathrm{effect}={\bar{\text{BCP}}}_{1}-\left(1/2\right){\bar{\text{P}}}_{1}-{\bar{\text{BCP}}}_{2}+\left(1/2\right){\bar{\text{P}}}_{2}$$

$$l=\text{Dominance}\times \mathrm{Dominance}\mathrm{effect}={\bar{\text{P}}}_{1}+{\bar{\text{P}}}_{2}+2{\bar{\text{F}}}_{1}+4{\bar{\text{F}}}_{2}-4{\bar{\text{BCP}}}_{1}-4{\bar{\text{BCP}}}_{2}$$

Where ${\bar{\text{P}}}_{1}$, ${\bar{\text{P}}}_{2}$, ${\bar{\text{F}}}_{1}$, ${\bar{\text{F}}}_{2}$, ${\bar{\text{BCP}}}_{1}$, and ${\bar{\text{BCP}}}_{2}$ are means of different generations, respectively. Furthermore, the variance component for each estimate was calculated as below:


$$\text{V}m=\text{V}\left({\bar{\text{F}}}_{2}\right)$$

$$\text{V}d=\text{V}\left({\bar{\text{BCP}}}_{1}\right)+\text{V}\left({\bar{\text{BCP}}}_{2}\right)$$

$$\text{V}h=\text{V}\left({\bar{\text{F}}}_{1}\right)+16\text{V}\left({\bar{\text{F}}}_{2}\right)+\left(1/4\right)\text{V}\left({\bar{\text{P}}}_{1}\right)+\left(1/4\right)\text{V}\left({\bar{\text{P}}}_{2}\right)+4V\left({\bar{\text{BCP}}}_{1}\right)+4V\left({\bar{\text{BCP}}}_{2}\right)$$

$$\text{V}i=4V\left({\bar{\text{BCP}}}_{1}\right)+4V\left({\bar{\text{BCP}}}_{2}\right)+16\text{V}\left({\bar{\text{F}}}_{2}\right)$$

$$\text{V}j=\text{V}\left({\bar{\text{BCP}}}_{1}\right)+\left(1/4\right)\text{V}\left({\bar{\text{P}}}_{1}\right)+\text{V}\left({\bar{\text{BCP}}}_{2}\right)+\left(1/4\right)\text{V}\left({\bar{\text{P}}}_{2}\right)$$

$$\text{V}l=\text{V}\left({\bar{\text{P}}}_{1}\right)+\text{V}\left({\bar{\text{P}}}_{2}\right)+4V\left({\bar{\text{F}}}_{1}\right)+16\text{V}\left({\bar{\text{F}}}_{2}\right)+16\text{V}\left({\bar{\text{BCP}}}_{1}\right)+16\text{V}\left({\bar{\text{BCP}}}_{2}\right)$$

where $\text{V}\,\left({\bar{\text{P}}}_{1}\right)$, $\text{V}\,\left({\bar{\text{P}}}_{2}\right)$, $\text{V}\,\left({\bar{\text{F}}}_{1}\right)$, $\text{V}\,\left({\bar{\text{F}}}_{2}\right)$, $\text{V}\,\left({\bar{\text{BCP}}}_{1}\right)$, and $\text{V}\,\left({\bar{\text{BCP}}}_{2}\right)$ were the variances of P_1_, P_2_, F_1_, F_2_, BCP_1_, and BCP_2_ generations, respectively.

The significance for the above genetic parameters was tested using the *t*-test. First, SE is worked out for each component separately by taking the square root of the variance of the respective component. The significance of the genetic effect is tested using the *t*-test in a similar manner as in the case of the scaling test. All these statistical analyses were conducted using the DOS-based Excel program, TNAUSTAT-Statistical package (Manivannan, 2014).

#### Heterosis

Mid-parent heterosis (MPH) was expressed as the percentage increase or reduction observed in the F_1_ progeny over that of the mid-parent value (Fonseca and Patterson, 1968). The residual heterosis over the mid-parent value (RHM) in the F_2_ generation was calculated as described by Rao (1980).


$$\mathrm{MPH}\left(\%\right)=\;\frac{\bar{{\text{F}}_{1}}-\bar{\text{MP}}}{\bar{\text{MP}}}\times 100$$

$$\mathrm{RHM}\left(\%\right)=\;\frac{\bar{{\text{F}}_{2}}-\bar{\text{MP}}}{\bar{\text{MP}}}\times 100$$

#### Heritability, Degree of Dominance, and Inbreeding Depression

Broad-sense heritability $\left[h_{b\,s}^{2}\right]$, narrow-sense heritability $\left[h_{n\,s}^{2}\right]$, and genetic advance (GA) from the heritability estimates were determined according to Warner (1952), whereas the percentage GA over the mean (GAM) was computed as described by Johnson et al. (1955). The degree of dominance, expressed as the square root of the ratio of dominance variance (H) to additive variance (D), was determined according to Robinson et al. (1949). Furthermore, the loss of fitness in progeny with reduced heterozygosity arising from consanguineous mating, known as inbreeding depression (ID), was estimated as described by Kempthrone (1957). Values were obtained using the following equations:


$$h_{b\,s}^{2}=\frac{{\text{VF}}_{2}-\left({\mathrm{VP}}_{1}+{\mathrm{VP}}_{2}+{\mathrm{VF}}_{1}\right)/3}{{\text{VF}}_{2}}\times 100$$

$$h_{n\,s}^{2}=\frac{2\times {\text{VF}}_{2}-\left({\mathrm{VBCP}}_{1}+{\mathrm{VBCP}}_{2}\right)}{{\text{VF}}_{2}}\times 100$$

$$\text{GA}=\frac{{\text{VF}}_{2}-\left(\mathrm{VE}\right)}{\mathrm{SQRT}\,\left({\mathrm{VF}}_{2}\right)}\times K$$

$$\text{GAM}=\frac{\text{GA}}{\text{Mean}\,\text{of}\,{\text{F}}_{2}}\times 100$$

$$\text{Degree}\,\text{of}\,\text{domicance}=\sqrt{H/D}$$

$$\text{Inbreeding}\,\text{depression}=\frac{\bar{{\text{F}}_{1}}-\bar{{\text{F}}_{2}}}{\bar{{\text{F}}_{1}}}\times 100$$

Where VP_1_, VP_2_, VF_1_, VBCP_1_, VBCP_2_, and VF_2_ are the variances of P_1_, P_2,_ F_1_, BCP_1_, BCP_2_, and F_2_, respectively; K is the selection differential, the value of which is 2.06 at a 5% selection intensity; and VE is the environmental variance.

## Results

**FIGURE 1:**
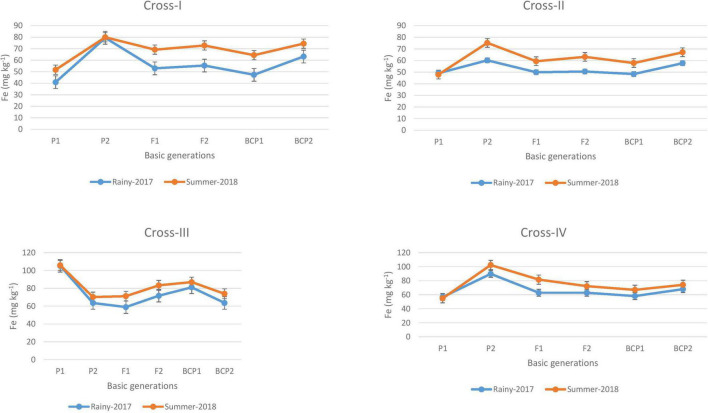
Mean performance of parents (P_1_ and P_2_), F_1_, F_2_ and backcross generations (BCP_1_ and BCP_2_) for grain iron (Fe) content among eight crosses during E_1_ (2017 rainy) and E_2_ (2018 summer) seasons.

**FIGURE 2:**
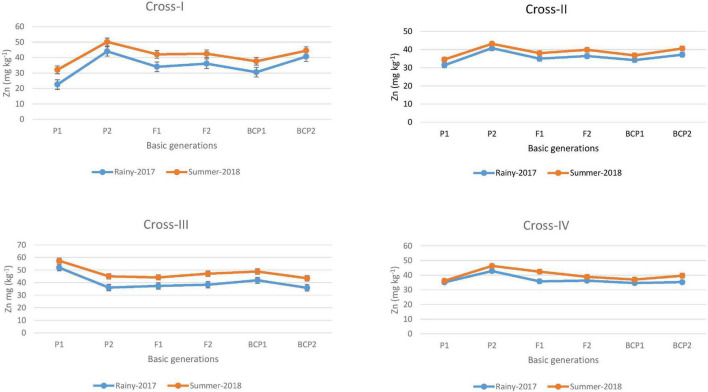
Mean performance of parents (P_1_ and P_2_), F_1_, F_2_ and backcross generations (BCP_1_ and BCP_2_) for grain zinc (Zn) content among eight crosses during E_1_ (2017 rainy) and E_2_ (2018 summer) seasons.

The ANOVA estimate for six generations of each cross revealed highly significant (*p* < 0.01) mean squares for grain Fe and Zn contents in E_1_ and E_2_, thereby indicating the presence of significant genetic variability for the two micronutrient traits (Table 3). Furthermore, analysis of the mean performance of each generation for both grain Fe and Zn contents revealed higher mean values in E_2_ than in E_1_ (Table 2 and Figures 1, 2), which was consistent among the four crosses. The frequency distribution of the number of plant samples for grain Fe and Zn contents in each generation (segregating) plotted during E_1_ and E_2_ is presented in Supplementary Figures 1–4.

**TABLE 3:** Analysis of variance for grain iron (Fe) and zinc (Zn) contents between the six generations within each cross evaluated in 2017 rainy (E_1_) and 2018 summer (E_2_).

Crosses	Generations		Replications		Error	
	df = 5		df = 2		df = 10	
	E_1_	E_2_	E_1_	E_2_	E_1_	E_2_
**Iron (Fe)**						
Cross I	548.72**	290.14**	0.13	31.89	1.83	9.68
Cross II	74.23**	2199.48**	0.89	4.79	1.93	2.74
Cross III	877.64**	552.20**	1.33	62.06	9.83	4.71
Cross IV	439.91**	769.97**	38.55**	3.02	3.91	3.91
**Zinc (Zn)**						
Cross I	173.20**	117.56**	0.06	9.66*	1.53	2.21
Cross II	29.01**	31.49**	9.46*	2.19	1.84	2.25
Cross III	131.37**	75.83**	6.33*	4.69	0.86	1.68
Cross IV	28.77**	42.86**	2.38	0.11	2.86	1.58

* and **, significant at 0.05 and 0.01 probability level; E_1_, 2017 rainy; E_2_, 2018 summer.

### Scaling Tests

Among all the crosses, at least one significant scaling test was observed out of four (A, B, C, and D) during E_1_ and E_2_ and this indicates the presence of epistasis or gene interactions except in cross-III during E_1_, wherein all the four scaling tests were non-significant (Table 4), which indicates that the simple additive and dominant model is adequate to explain the cause of variation in grain Fe and Zn contents.

**TABLE 4:** Scaling tests for grain iron (Fe) and zinc (Zn) contents of different cross evaluated in 2017 rainy (E_1_) and 2018 summer (E_2_).

Crosses	Scaling tests							
	E_1_				E_2_			
	A	B	C	D	A	B	C	D
	
	Fe							
Cross I	0.79NS	−5.97*	−4.8NS	0.19NS	7.86**	−0.33NS	21.23**	6.85**
Cross II	−2.61 NS	4.43*	−7.12NS	−4.47*	8.38**	−0.35NS	10.77*	1.37NS
Cross III	−1.94 NS	4.73NS	0.06NS	−1.37NS	−3.05NS	6.27*	14.88**	5.83*
Cross IV	−3.32 NS	−16.6**	−20.7**	−0.35NS	−2.36NS	−35.7**	−31.5**	3.25NS
	
	**Zn**							
	
Cross I	4.39**	3.23NS	9.55**	0.97NS	1.13NS	−3.28NS	3.51NS	2.83*
Cross II	2.07NS	−1.48NS	3.65NS	1.53NS	0.93NS	−0.02NS	5.59NS	2.34NS
Cross III	−5.52*	1.28NS	−6.33NS	−1.05NS	−3.93NS	−2.17NS	−2.37NS	1.86NS
Cross IV	−1.65NS	−8.1**	−4.45NS	2.65NS	−4.55**	−9.4**	−11.8**	1.10NS

* and **, significant gene effect at 0.05 and 0.01 probability level; E_1_, 2017 rainy; E_2_, 2018 summer; NS, non significant.

### Genetic Effects

With respect to the grain Fe content, partitioning of the generation mean into six different genetic components revealed that the effect of the mean was significant (*p* < 0.01), and its magnitude was higher than that of the other genetic effects assessed, namely additive (d) and dominant (h), and the three interaction effects, additive × additive (i), additive × dominant (j), and dominant × dominant (l), among the four crosses studied. The cross-wise direct and inter-allelic interaction effects for the grain Fe content in cross-I showed the presence of a significant additive genetic effect (*p* < 0.01) and an additive × dominant interaction effect (*p* < 0.05) in E_1_ (Table 5). In E_2_, both additive (*p* < 0.01) and dominant (*p* < 0.01) genetic effects were significant, wherein the magnitude of the additive genetic effect was 1% higher than that of the dominant genetic effect. Among the interaction effects, additive × additive and additive × dominant effects were significant (*p* < 0.01), wherein the magnitude of the additive × additive genetic effect was 70% higher than that of the additive × dominant effect. In cross-II, the additive (*p* < 0.01) genetic effect along with additive × additive (*p* < 0.05) and additive × dominant (*p* < 0.05) interaction effects were significant in E_1_. The magnitude of the additive × additive genetic effect was 61% higher than that of the additive × dominant effect. In E_2_, only additive genetic effect (*p* < 0.01) and the additive × dominant (*p* < 0.05) interaction effect were significant. In cross-III in E_1_, all the four scaling tests were non-significant that indicate the absence of epistasis. Nevertheless among direct genetic effects, both additive and dominant effects were significant (*p* < 0.01), wherein the magnitude of the dominant genetic effect was 24% higher than that of the additive. In E_2_, both additive and dominant genetic effects along with additive × additive and additive × dominant interaction effects were significant (*p* < 0.05), wherein the magnitude of the dominant genetic effect was 54% higher than that of the additive effect, and the magnitude of the additive × additive was 60% higher than that of the additive × dominant interaction effect. Cross-IV in E_1_ was characterized by significant additive (*p* < 0.01) and dominant (*p* < 0.05) genetic effects with a higher magnitude of an additive genetic effect. Among interaction effects, additive × dominant and dominant × dominant were significant (*p* < 0.01), wherein the magnitude of dominant × dominant was 65% higher than that of the additive × dominant. The contrasting directions of the dominant genetic effect and dominant × dominant interaction effects indicated the presence of duplicate epistasis. In E_2_, an additive genetic effect along with additive × dominant and dominant × dominant interaction effects were significant (*p* < 0.01), wherein the magnitude of the dominant × dominant interaction effect was 63% higher than that of the additive × dominant.

**TABLE 5:** Direct and interaction gene effects for grain iron (Fe) and zinc (Zn) contents in different cross evaluated 2017 rainy (E_1_) and 2018 summer (E_2_).

Iron (Fe)														
**Crosses**	**E_1_**							**E_2_**						
		
	**m**	**d**	**h**	**i**	**j**	**l**	**E**	**m**	**d**	**h**	**i**	**j**	**l**	**E**
Cross-I	55**	−15.88**	–7.64	–0.38	3.38*	5.56	–	73**	−10.45**	−10.13*	−13.70**	4.09**	6.17	–
Cross-II	51**	−9.05**	4.21	8.95*	−3.52*	–10.77	–	63**	−9.27**	–4.19	–2.74	4.37*	–5.29	–
Cross-III	71**	17.53**	−22.80**	2.73	–3.31	–5.52	–	83**	13.14**	25.82**	−11.65*	−4.66*	8.43	–
Cross-IV	63**	−9.88**	−9.70*	0.7	6.65**	19.24**	D	72**	−7.14**	–3.64	–6.51	16.66**	44.54**	–

**Zinc (Zn)**														

Cross-I	36**	−10.12**	–1.17	–1.93	0.58	–5.69	–	42**	−6.83**	–4.69	−5.66*	2.21**	7.81	–
Cross-II	36.46	−2.92**	–4.15	–3.05	1.77	2.46	–	39.86**	−3.85**	–5.47	–4.67	0.47	3.75	–
Cross-III	38**	5.97**	–3.07	2.1	−3.4**	2.14	–	47.05**	5.28**	−10.76**	–3.72	–0.88	9.82	–
Cross-IV	36**	–0.61	−8.47**	–5.3	3.23**	15.05**	D	39**	−2.64**	–1.00	2.2	2.43**	16.15**	–

* and **, significant gene effect at 0.05 and 0.01 probability level.; m, mean; d, additive; h, dominance; i, additive × additive; j, additive × dominant; l, dominant × dominant; E, epistasis; D, duplicatory; C, complementary; E_1_, 2017 rainy; E_2_, 2018 summer; NI, no interaction.

The mean effect for the Zn content in grains indicated a significant (*p* < 0.01) predominant effect compared with all other assessed genetic effects, including the different interaction effects. The cross-wise direct genetic and interaction effects in E_1_ and E_2_ for the grain Zn content revealed that in cross-I, the additive genetic effect was significant (*p* < 0.01) in E_1_ and E_2_. Among interaction effects only in E_2_, the additive × additive (*p* < 0.05) and additive × dominant (*p* < 0.01) interaction effects were significant, wherein the magnitude of the additive × additive effect was 61% higher than that of the additive × dominant effect. In cross-II, only the additive genetic effect was significant (*p* < 0.01) in E_1_ and E_2_. All three interaction effects were non-significant, both in E_1_ and E_2_, indicating an absence of epistasis. In cross-III in E_1_, the additive genetic effect along with additive × dominant interaction effect (*p* < 0.01) was significant (*p* < 0.01). In E_2_, all four scaling tests were found to be non-significant, indicating the absence or lack of role of epistasis. Nevertheless among the direct genetic effects, both additive and dominant genetic effects were significant (*p* < 0.01), wherein the magnitude of the dominant genetic effect was 51% higher than that of the additive genetic effect in E_2_. Unlike in earlier crosses, non-significant additive and significant (*p* < 0.01) dominant genetic effect was observed in cross-IV in E_1_. Among the interaction effects, the additive × dominant (*p* < 0.01) and dominant × dominant (*p* < 0.01) were significant, wherein the magnitude of the dominant × dominant was 79% higher than that of the additive × dominant. The contrasting directions of dominant genetic and dominant × dominant interaction effects provided evidence for the presence of duplicate epistasis in E_1_. In contrast to E_1_, we detected a significant additive genetic effect, along with significant additive × dominant (*p* < 0.05) and dominant × dominant (*p* < 0.01) interaction effects in E_2_, wherein the magnitude of the dominant × dominant effect was 85% higher than that of additive × dominant effect.

### Heritability and Percentage Genetic Advance Over the Mean

A high broad-sense heritability was recorded for the grain Fe content among all the four crosses in E_1_ and E_2_. Furthermore, we also detected high narrow-sense heritability among four crosses with a magnitude varying from 72.14 to 86.64% in E_1_ (Table 6). Similarly, in E_2_, we recorded high narrow-sense heritability for the four crosses, among which the magnitude varied from 61.78 to 90.50%. High GAM was recorded for all crosses in E_1_ and E_2_, wherein the magnitude varied from 34.50 to 48.42% and from 25.46 to 40.34% in E_1_ and E_2_, respectively.

**TABLE 6:** Estimates of genetic components for grain iron (Fe) and zinc (Zn) contents in different crosses evaluated in 2017 rainy (E_1_) and 2018 summer (E_2_).

Crosses	GCV%		PCV%		$h_{b\,s}^{2}\%$		$h_{n\,s}^{2}\%$		GAM%		H/D		ID	
	E_1_	E_2_	E_1_	E_2_	E_1_	E_2_	E_1_	E_2_	E_1_	E_2_	E_1_	E_2_	E_1_	E_2_
**Iron (Fe)**														
Cross I	33.28	25.48	33.71	26.08	94.04	91.21	74.83	90.50	43.75	33.34	0.72	0.13	–4.60	–5.24
Cross II	36.69	31.50	37.28	32.47	92.67	88.01	72.14	80.18	48.42	40.34	0.75	0.44	–1.17	–6.36
Cross III	33.02	30.97	33.78	31.69	91.12	90.29	86.64	77.32	46.29	39.40	0.32	0.58	–21.69	–17.07
Cross IV	25.17	20.80	26.21	21.91	84.71	78.87	79.12	61.78	34.50	25.46	0.38	0.74	–0.06	11.41
**Zinc (Zn)**														
Cross I	33.44	22.18	34.95	24.27	80.75	68.89	57.18	59.04	37.00	24.49	0.91	0.58	–5.88	–0.93
Cross II	37.30	34.84	38.97	36.38	80.52	79.97	55.06	48.76	41.77	37.50	0.96	1.13	–4.18	–4.72
Cross III	41.98	37.43	42.84	38.60	89.97	83.31	58.58	35.15	51.76	40.10	1.04	1.66	–2.68	–6.62
Cross IV	33.28	26.47	34.16	28.37	86.57	68.45	48.55	30.60	37.99	26.38	1.25	1.57	–1.32	8.34

*E_1_, 2017 rainy; E_2_, 2018 summer; GCV, genetic coefficient of variation; PCV, phenotypic coefficient of variation; $h_{b\,s}^{2}$, heritability broad sense; $h_{n\,s}^{2}$, heritability narrow sense; GAM, genetic advance percent over mean; H/D, degree of dominance; ID, inbreeding depression.*

Similarly, we recorded high broad-sense heritability values for Zn among all crosses in E_1_ and E_2_, whereas a moderate narrow-sense heritability with magnitudes ranging from 48.55 to 58.58% and from 30.60 to 59.04% was recorded for all crosses in E_1_ and E_2_, respectively. Moreover, high GAM was recorded among all the crosses in E_1_ and E_2_, with magnitudes ranging from 37.00 to 51.76% and from 24.49 to 40.10% in E_1_ and E_2_, respectively.

### Heterosis, Degree of Dominance, and Inbreeding Depression

Significant (*p* < 0.01) negative MPH was recorded among all the four crosses for the grain Fe content with the highest being obtained for cross-III (−30.20%) and the lowest for cross-II (−8.90%) in E_1_ (Table 7). Similarly, among all crosses, RHM was significant (*p* < 0.01) for the grain Fe content in a negative direction, wherein the magnitude was the highest for cross-III (−15.14%) and the lowest for cross-II (−7.66%) in E_1_. In E_2_, a significant (*p* < 0.01) MPH was recorded only in cross-III (−19.36%). Surprisingly, we established MPH estimates in a positive direction for crosses I and IV, but the effects were non-significant. RHM in E_2_ was significant only among crosses III and IV, wherein the magnitude of RHM was the highest for cross-IV (−9.70%) and the lowest for cross-II (−0.43%). Furthermore, it was observed that except cross-I, the rest of the three crosses recorded RHM in negative direction in E_2_. None of the F_1_ populations exceeded the respective better parent in either E_1_ or E_2_. Furthermore, we detected no significant ID among the crosses in E_1_ and E_2_. The degree of dominance among the four crosses varied from as low as 0.32 for cross-III to as high as 0.75 for cross-II in E_1_ and from 0.13 for cross-I to 0.74 for cross-IV in E_2_.

**TABLE 7:** Heterosis components for grain iron (Fe) and zinc (Zn) contents for different crosses evaluated in 2017 rainy (E_1_) and 2018 summer (E_2_).

Iron (Fe)					Zinc (Zn)			
Crosses	MP		RHM		MP		RHM	
	E_1_	E_2_	E_1_	E_2_	E_1_	E_2_	E_1_	E_2_
Cross I	−12.12**	5.18	−8.10**	4.91	1.95	2.22	8.17*	3.19
Cross II	−8.90**	–3.45	−7.66**	–0.43	–3.05	–2.00	0.93	2.59**
Cross III	−30.20**	−19.36**	−15.14**	−4.01*	−12.28**	−13.53**	−9.91**	−7.86**
Cross IV	−14.05**	3.52	−13.72**	−9.70**	−8.22*	2.78	−6.98*	−5.79*

* and **, significant at 0.05 and 0.01 probability level; E_1_, 2017 rainy; E_2_, 2018 summer; MP, mid parent heterosis; RHM, relative heterosis over mid-parent.

With respect to grain Zn content, we detected a significant MPH for crosses III (*p* < 0.01) and IV (*p* < 0.05). Among four crosses, the highest magnitude of MPH was recorded in cross-III (−12.28%) and the lowest in cross-I (1.95%), although the difference was not significant. With the exception of cross-I, the recorded MPH for rest of the crosses was in a negative direction in E_1_. Furthermore, among the four crosses in E_1_, we recorded a significant (*p* < 0.01) RHM for crosses I, III, and IV, with the highest magnitude being recorded in cross-III (−9.91%) and the lowest in cross-II (0.93%) which was found to be non-significant. However, the RHM was observed to be in a positive direction for crosses I and II, whereas it was in a negative direction for crosses III and IV. In E_2_, a significant (*p* < 0.01) MPH was recorded only in cross-III. The magnitude of MPH was found to be the highest in cross-III (−13.53%) and the lowest in cross-II (−2.00%), the difference between which was non-significant. Furthermore, MPH recorded was in a positive direction for crosses I and IV, whereas, it was in a negative direction for crosses II and III. RHM was significant (*p* < 0.01) among crosses II, III, and IV, wherein the highest magnitude was recorded in cross-III (−7.86%) and the lowest in cross-II (2.59%). As in E_1_, the RHM recorded for crosses I and II was in a positive direction, and that for crosses III and IV was in a negative direction in E_2_. None of the F_1_ populations performed better than the better parent in E_1_ and E_2_, thereby indicating an absence of BPH. In both E_1_ and E_2_, we detected no significant ID among the assessed crosses. Furthermore, the degree of dominance estimates among the four crosses varied from as low as 0.91 for cross-I to as high as 1.25 for cross-IV in E_1_ and from 0.58 for cross-I to 1.66 for cross-III in E_2_.

### Correlations Between Fe and Zn

With respect to correlations between Fe and Zn contents in pearl millet grain, we performed Pearson’s correlation analysis to assess the different generations among each cross in each of the two environments (seasons) (Table 8) and accordingly detected highly significant positive correlations between grain Fe and Zn contents among all crosses in E_1_ and E_2_. The only exception in this regard was cross-I in E_1_, for which the positive correlation was non-significant. Among the four crosses, the magnitude of the correlation between grain Fe and Zn contents was the highest in cross-II in both E_1_ (*r* = 0.692, *p* < 0.01) and E_2_ (*r* = 0.674, *p* < 0.01). A similar positive correlation was observed between grain Fe and Zn contents in backcross populations, with the exception of cross-III (*r* = − 0.089). Highly significant positive correlations (*p* < 0.01) between grain Fe and Zn contents were also detected for all three segregating generations (F_2_, BCP_1_, and BCP_2_) in E_1_ and E_2_, with the magnitude of correlation being the lowest in cross-IV in both E_1_ (*r* = 0.168, *p* < 0.01) and E_2_ (*r* = 0.371, *p* < 0.01).

**TABLE 8:** Correlation between grain iron and zinc contents in F_2_, BCP_1_, BCP_2_ within individual crosses and across all the generation within individual crosses evaluated in 2017 rainy (E_1_) and 2018 summer (E_2_).

Sr No	Crosses	Correlation between Fe and Zn							
		Within generation						Across all generations	
		F_2_		BCP_1_		BCP_2_		F_2_, BCP_1_ and BCP_2_	
		E_1_	E_2_	E_1_	E_2_	E_1_	E_2_	E_1_	E_2_
1	Cross-I	0.069	0.637**	0.098	0.061	0.001	0.253**	0.246**	0.537**
2	Cross-II	0.692**	0.674**	0.077	0.513**	0.633**	0.605**	0.532**	0.644**
3	Cross-III	0.469**	0.570**	–0.089	0.146	0.212*	0.198**	0.348**	0.454**
4	Cross-IV	0.256**	0.493**	0.083	0.070	0.018	0.158	0.168**	0.371**

* and **, significant at 0.05 and 0.01 probability level; E_1_, 2017 rainy; E_2_, 2018 summer.

## Discussion

The ANOVA revealed significant generation means for grain Fe and Zn contents among all the four crosses in E_1_ and E_2_, thereby indicating a substantial degree of genetic variability for grain Fe and Zn contents among segregating populations obtained from crossing contrasting parents. The mean performances of parents and F_1_ progeny revealed a slight increase in the levels of Fe and Zn contents in the grains of pearl millet cultivated in the second (E_2_) season of the present study compared with the first (E_1_) season. This is in line with the expected observation, given that the accumulation of Fe and Zn contents in grains is predicted to be higher in the summer environment due to the higher absorption from roots in response to higher rates of transpiration. Moreover, in the case of the grain Fe content, we established that the levels of available Fe in soil were higher in E_2_ (5.0 mg kg^–1^ Fe). Interestingly though the available Zn in soil was relatively low, this was not reflected in the grain Zn content in E_2_; thus, the mean performance with respect to the grain Zn content would probably be stable in E_1_ and E_2_. Previous studies have likewise reported similar patterns of grain Fe and Zn contents in pearl millet during rainy and summer seasons (Govindaraj et al., 2013; Kanatti et al., 2014). In the present study, we recorded differences in the grain levels of Fe and Zn of 1 to 15 mg kg^–1^ and 1 to 9 mg kg^–1^, respectively, between parents cultivated in the two environments (E_1_ and E_2_), whereas we found that the mean F_2_ distribution was normally distributed for all crosses (Supplementary Figures 1–4).

Epistasis is defined as any form of non-allelic interaction (Phillips, 1998; Boubacar et al., 2020). In this respect, comprehensive knowledge of gene action and interactions can contribute to determining the selection of breeding methods that efficiently exploits the genetic variance, which in turn can assist in interpreting the role of breeding systems in crop evolution (Hallauer and Miranda, 1981). The occurrence of significant epistasis has a tendency to bias the estimation of variance components. For example, polygenic inheritance models consisting of a large array of allelic interactions would tend to cause underestimations or overestimations of heritability, generally of the narrow-sense type, that in turn would contribute to additional bias in predicted gains. Generation mean analysis thus involves several basic generations from crosses between two inbred lines and provides estimates of epistatic effects. To assess these effects, we adopted the six-parameter model described by Hayman (1958) in the present study and used this to examine four crosses.

Significant additive genetic effects were consistently recorded for the grain Fe content among all the four crosses in each of the two assessed environments. The higher magnitude of the additive genetic effect over the dominant effect among the crosses (I, II, and IV) indicates that the genes underlying the grain Fe content in pearl millet are predominantly governed by additive genetic effects. Similarly, with the exception of cross-IV, highly significant additive genetic effects among the crosses were detected for the Zn content in grains in E_1_ and among all the crosses in E_2_, thus indicating that additive genetic effects also play a major role in determining the inheritance of genes underlying grain Zn content. It is important to note that in the present study, the negative value for “*d*” (additive) indicates that the parent with lower grain Fe and Zn contents was selected as P_1_, and the parent with a higher grain Fe content was selected as P_2_. Hence, when comparing the magnitude of genetic effects, absolute values were taken into consideration. In this regard, the findings of a previous study have also indicated that the positive or negative direction of additive and dominant genetic effects signifies that it is the parent with the highest number of genes or positive alleles that contributes to the increment in the trait of interest (Parihar et al., 2016). Similar predominant significant additive genetic effects for grain Fe and Zn contents have also been previously reported in pearl millet (Velu et al., 2011; Govindaraj et al., 2013; Kanatti et al., 2014), sorghum (Kiran, 2013; Kumar et al., 2013; Hariprasanna et al., 2014; Gaddameedi et al., 2018), maize (Gorsline et al., 1964; Long et al., 2004; Chen et al., 2007; Chakraborti et al., 2010), and rice (Zhang et al., 2004).

With respect to the grain Fe content, we detected a high narrow-sense heritability for all the four crosses in E_1_ and E_2_, thereby providing further evidence that the genes underlying the grain Fe content are largely under additive genetic control. Similarly, moderate-to-high narrow-sense heritability has also been previously reported for the grain Fe content in pearl millet (Velu et al., 2011). Contrastingly, the narrow-sense heritability recorded for the grain Zn content was either low or, in the majority of cases, it was moderate. In addition, the absence of BPH, along with non-significant ID, for all the four crosses tends to be compatible with the additive genetic effect for grain Fe and Zn contents. Notably, the non-significant ID detected for Fe and Zn contents in grains in the present study is consistent with the findings of a previous study on pearl millet (Rai et al., 2017). The degree of dominance for the grain Fe content was negligible, whereas that for the grain Zn content was either close to or higher than one during E_1_ and E_2_, indicating the some role of partial dominance as non-additive genetic effects. Complementary to this trend in some of the hybrids in the present study, the MPH effect for the grain Zn content was observed to be in a negative direction in E_1_ and E_2_, thereby indicating that the effects of the genes determining lower Zn levels are partially dominant. Moreover, it is also probable that the effects of genes acting additively for grain Zn are influenced by genetic background, particularly in a negative direction, resembling the low levels of partial dominance. Similar significant negative MPH has also been reported in previous studies on pearl millet (Velu et al., 2011; Rai et al., 2012; Govindaraj et al., 2013; Kanatti et al., 2014).

The significance of one or more scaling tests for the grain Fe content among most of the crosses in E_1_ and among all the crosses in E_2_ provides evidence for the role of epistatic interaction effects in the inheritance genes governing the grain Fe content in pearl millet. Among such interactions, additive × dominant was consistently significant across E_1_ and E_2_. Similar results have been reported in previous studies for the grain Fe content in rice (Chamundeswari, 2010) and maize (Chakraborti et al., 2010). However, in the case of cross-II in E_1_ and crosses I and III in E_2_, we found the role of additive × additive interactions for the grain Fe content to be significant. Additive genetic effects along with the additive × additive interaction effects represent fixable genetic variance, and hence the progeny *per se* selection would presumably be effective for enhancing the grain Fe content in pearl millet. Similar significant additive × additive genetic effects for the grain Fe content have been previously reported in pearl millet (Boubacar et al., 2020), rice (Mallimar et al., 2017), and maize (Chakraborti et al., 2010). We suspect that most of these interaction effects would be cross-specific and would show a slight environmental variation for the same cross, whereas direct gene effects (additive) would be relatively consistent.

With the exception of additive × dominant effects for cross-III in E_1_, we found that the epistatic interaction effects for the grain Zn content were non-significant for crosses II and III in E_1_ and E_2_, wherein their magnitudes were also relatively lower than those of the respective additive genetic effects. This further indicates that among these two crosses, the grain Zn content is predominantly governed by additive genetic effects, and at most, interaction effects play only a rudimentary role. In cross-I, all three of the assessed interaction effects showed a non-significant effect in E_1_, whereas in E_2_, both additive × additive and additive × dominant interaction effects showed a significant effect. Moreover, the magnitude of the additive × additive interaction effect was almost double that of the additive × dominant effect. The significant additive genetic effect coupled with the predominant additive × additive interaction effect would tend to indicate that the inheritance genes governing the grain Zn content are largely governed by additive genetic effects in cross-I. We found that the interaction effects associated with grain Zn content, such as additive × dominant and dominant × dominant, were consistently significant in both E_1_ and E_2_ in the case of cross-IV, thereby further confirming that these interaction effects are cross-specific. A similar higher magnitude of the dominant × dominant interaction effect for the grain Zn content has also been previously reported in sorghum (Kiran, 2013) and rice (Chamundeswari, 2010; Gajanan, 2015; Rani, 2016). Moreover, we found that only cross-IV in E_1_ showed a significant dominant genetic effect along with a dominant × dominant interaction effect, which was in the opposite direction (sign), indicating that the grain Zn content is governed by duplicate epistasis. However, the non-significant effects of the dominant × dominant interactions among crosses I, II, and III precluded a determination of the respective types of epistasis. The duplicate type of epistasis found for both Fe and Zn in cross-IV in E_1_ suggests that the selection for high grain Fe and Zn recombinants is possible through pedigree breeding by delaying the selection to later generations. Similar duplicate epistatic effects have previously been reported for Fe and Zn in sorghum (Kiran, 2013; Gaddameedi et al., 2018) and rice (Chamundeswari, 2010; Gajanan, 2015; Rani, 2016; Mallimar et al., 2017). In contrast, the consistent absence of a significant interaction effect and predominant additive genetic effect in E_1_ and E_2_ for both grain Fe and Zn content indicates that they are under the control of a simple digenic inheritance, which is similar to the simple digenic inheritance for the grain Zn content previously reported in pearl millet by Kumar et al. (2018).

Notably, none of the hybrid progeny obtained in the present study exceeded the mean of the better parent for the grain contents of Fe and Zn, which implies that there was no BPH, and in turn, indicates that the predominance of additive components for grain Fe and Zn inheritance would make it difficult to obtain BPH based on genetic variance. Furthermore, in most instances, we observed a significant RHM for both grain Fe and Zn contents in E_1_ and E_2_, although for almost all the hybrids, this was in a negative direction, indicating that the MPH obtained in the F_1_ generation was retained in F_2_ populations. Moreover, it would tend to imply that even in the F_2_ progeny, the expression of grain Fe and Zn contents could be masked by the undesirable linkage of partially dominant genes (negative alleles) that govern the low levels of Fe and Zn contents in grains. Similar negative MPH and partial dominance have been detected previously in pearl millet (Govindaraj, 2006; Velu et al., 2011). In general, the correlation between grain Fe and Zn contents was positive and significant, and the positive direction observed in hybrids and segregating generations in the present study provides evidence of the co-segregation of these two micronutrients. Thus, these findings tend to indicate that the grain Fe content in pearl millet could be simultaneously enhanced with the grain Zn content as an associated trait, which would be consistent with previous reports of a positive correlation between grain Fe and Zn contents in pearl millet (Gupta et al., 2009; Velu et al., 2009; Rai et al., 2012, 2014; Govindaraj et al., 2013).

## Conclusion

In summary, the generation mean analysis revealed that with additive genetic variance, additionally, there are gene interaction contributions indicating additive × additive and additive × dominant interaction effects for grain Fe and Zn contents in pearl millet. This study confirms the significant role of additive gene effects in grain Fe and Zn improvement reported earlier in pearl millet, whereas there is a chance of specific gene interactions exist merits further strategic genetic and genomic studies using more inbreds for a profound understanding of biofortified hybrid breeding. Among the direct genetic effects, additive genetic effects predominantly govern its evidence of grain Fe and Zn contents. Therefore, duplicate epistatic interactions would not constitute a substantial impediment to the improvement of grain Fe and Zn contents in pearl millet, which could be overcome by practicing early selection (F_2_s) for agronomic traits and delayed selection (>F_3_−F_4_s) in Fe and Zn trait mainstreaming breeding pipelines.

## Data Availability Statement

The datasets presented in this study can be found in online repositories. The names of the repository/repositories and accession number(s) can be found in the article/Supplementary Material.

## Author Contributions

MG conceptualized the research and contributed to the experimental materials. MG, SG, and MP designed the experiments. MP executed the field/laboratory experiments and data collection. MP, AK, and MG interpreted the analysis of data. MP, MG, AK, SG, TG, BD, and KS prepared the manuscript. All authors contributed to the article and approved the submitted version.

## Conflict of Interest

The authors declare that the research was conducted in the absence of any commercial or financial relationships that could be construed as a potential conflict of interest.

## Publisher’s Note

All claims expressed in this article are solely those of the authors and do not necessarily represent those of their affiliated organizations, or those of the publisher, the editors and the reviewers. Any product that may be evaluated in this article, or claim that may be made by its manufacturer, is not guaranteed or endorsed by the publisher.

## Footnotes

1
https://www.who.int/health-topics/anaemia
