# Supplementary material for: Generation Mean Analysis Reveals the Predominant Gene Effects for Grain Iron and Zinc Contents in Pearl Millet
Source: Front Plant Sci. 2022 Jan 28;12:693680. doi: 10.3389/fpls.2021.693680 (PMC8831551; doi:10.3389/fpls.2021.693680)
Supplement: Supplementary file 1 [file Data_Sheet_1.docx]

| **Supplementary Table 1. Weather report: 2017 Rainy (E_1_), ICRISAT, Patancheru-India** | | | | | |
| --- | --- | --- | --- | --- | --- |
| **Name of Month** | **Rainfall**  **(mm)** | **Max Temp**  **(^o^C)** | **Min Temp**  **(^o^C)** | **Relative Humidity at 7:17 Hr. (%)** | **Relative Humidity at 14:17 Hr. (%)** |
| Jun | 229.8 | 33.11 | 23.64 | 85.00 | 62.75 |
| Jul | 156.5 | 30.60 | 22.42 | 85.9 | 65.51 |
| Aug | 256.8 | 29.23 | 22.10 | 89.18 | 71.79 |
| Sep | 183.6 | 30.71 | 22.32 | 91.15 | 63.96 |
| Oct | 281.8 | 30.87 | 20.53 | 91.21 | 58.47 |

| **Supplementary Table 2. Weather report: 2018 summer (E_2_), ICRISAT, Patancheru-India** | | | | | |
| --- | --- | --- | --- | --- | --- |
| **Name of Month** | **Rainfall**  **(mm)** | **Max Temp**  **(^o^C)** | **Min Temp**  **(^o^C)** | **Relative Humidity at 7:17 Hr. (%)** | **Relative Humidity at 14:17 Hr. (%)** |
| Feb | 0.00 | 31.46 | 14.10 | 73.75 | 26.67 |
| Mar | 0.40 | 35.93 | 18.32 | 64.15 | 22.51 |
| Apr | 27.69 | 37.52 | 22.00 | 70.20 | 31.19 |
| May | 21.30 | 39.45 | 24.67 | 66.64 | 30.87 |
