# Supplementary figures and images for: Generation Mean Analysis Reveals the Predominant Gene Effects for Grain Iron and Zinc Contents in Pearl Millet
Source: Front Plant Sci. 2022 Jan 28;12:693680. doi: 10.3389/fpls.2021.693680 (PMC8831551; doi:10.3389/fpls.2021.693680)

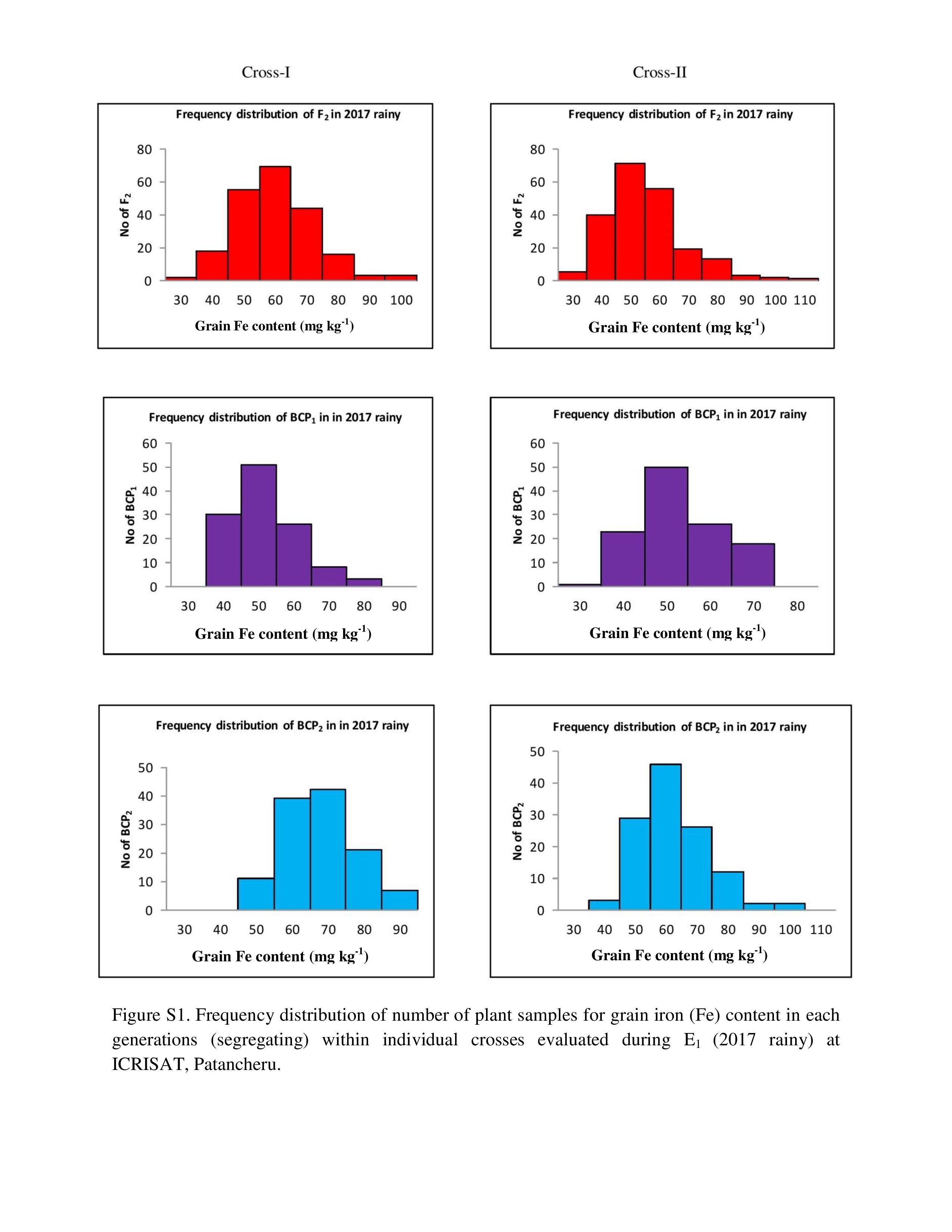

Supplement: Supplementary file 2 [file Image_1.JPEG]

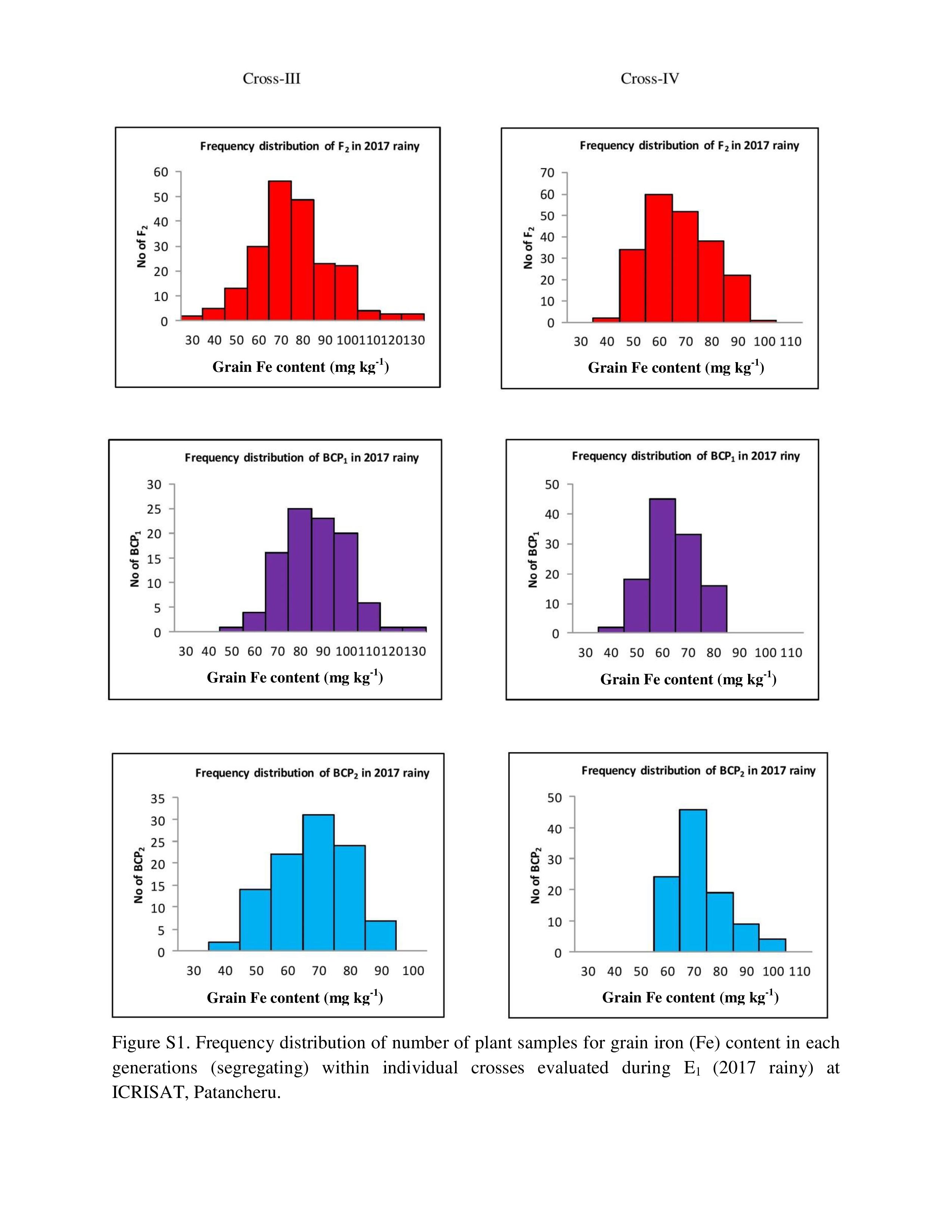

Supplement: Supplementary file 3 [file Image_2.JPEG]

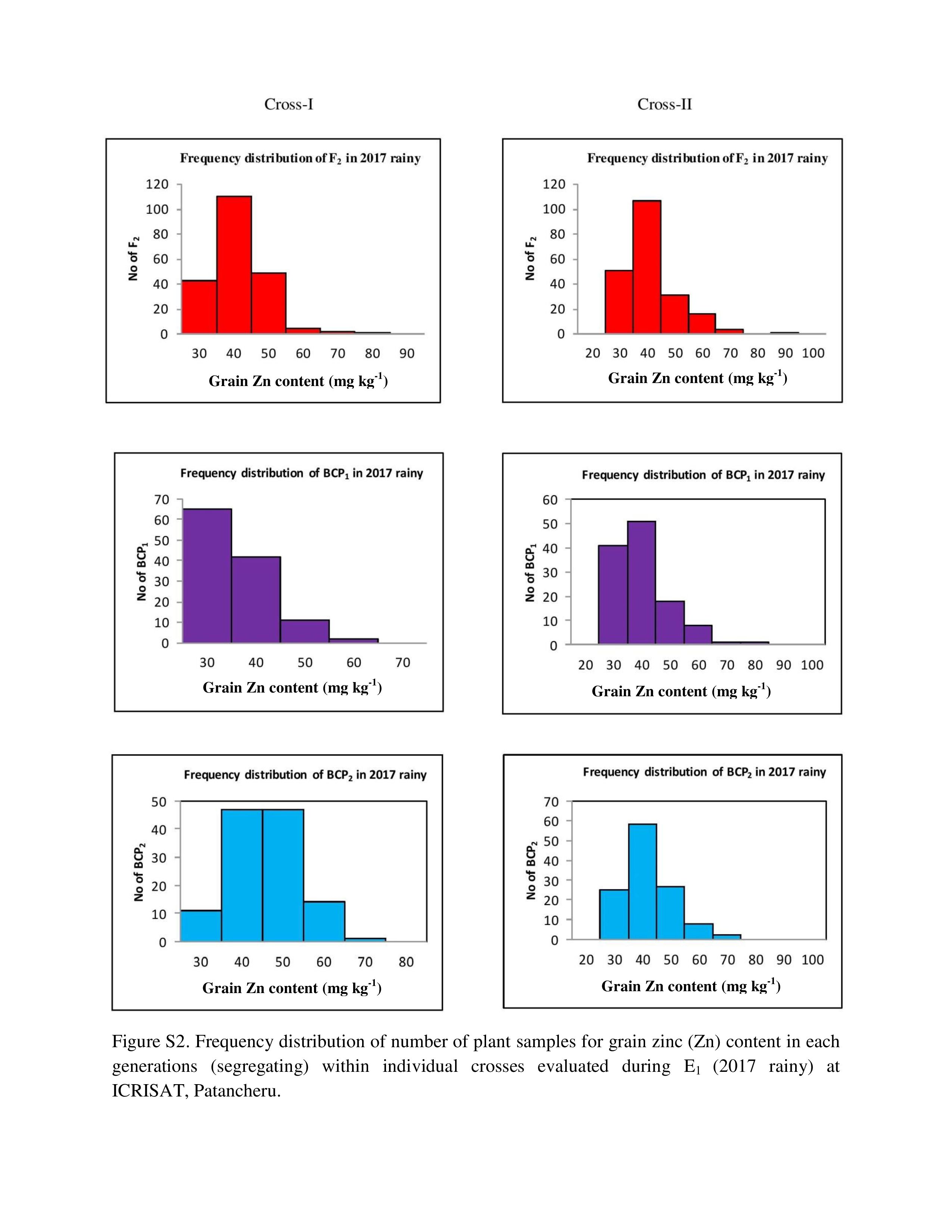

Supplement: Supplementary file 4 [file Image_3.JPEG]

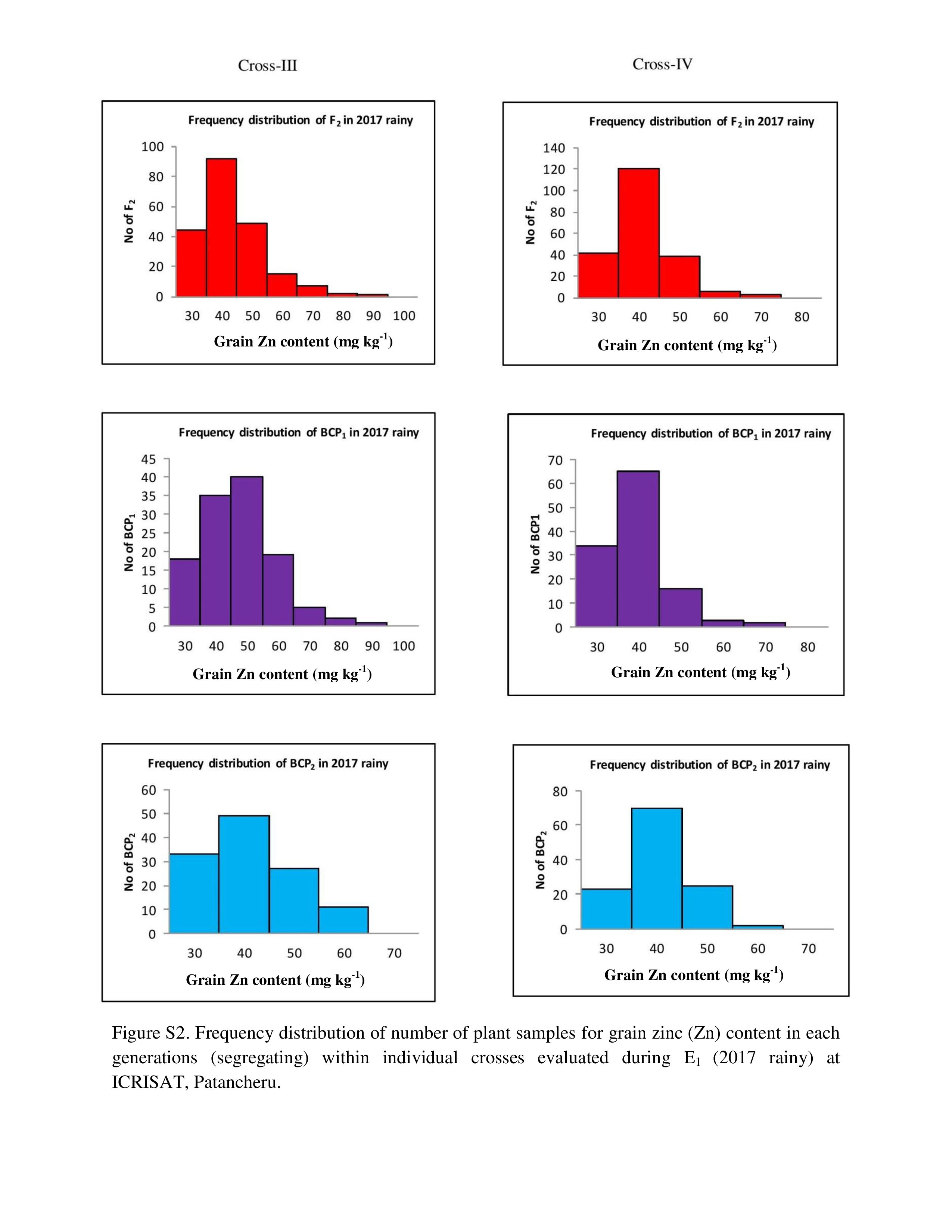

Supplement: Supplementary file 5 [file Image_4.JPEG]

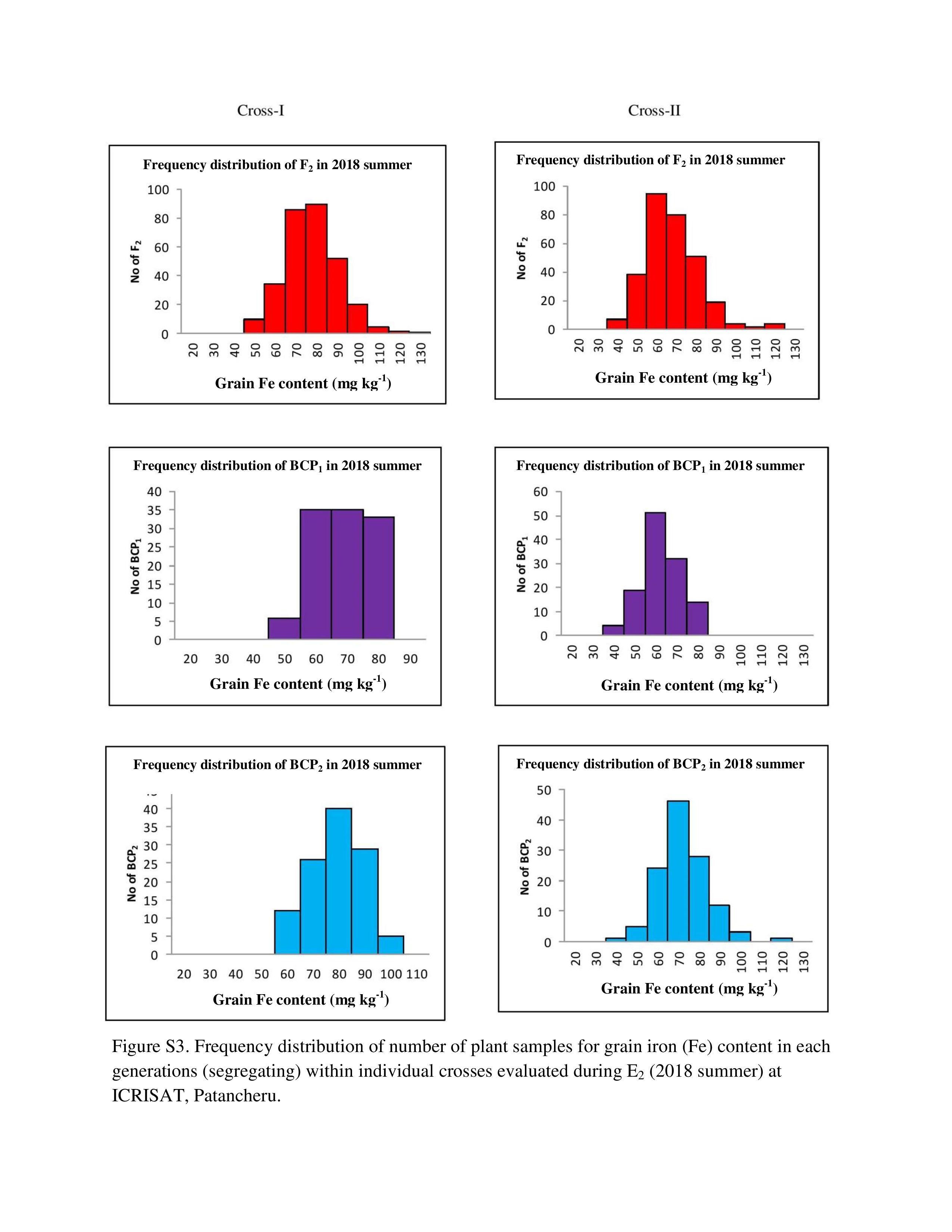

Supplement: Supplementary file 6 [file Image_5.JPEG]

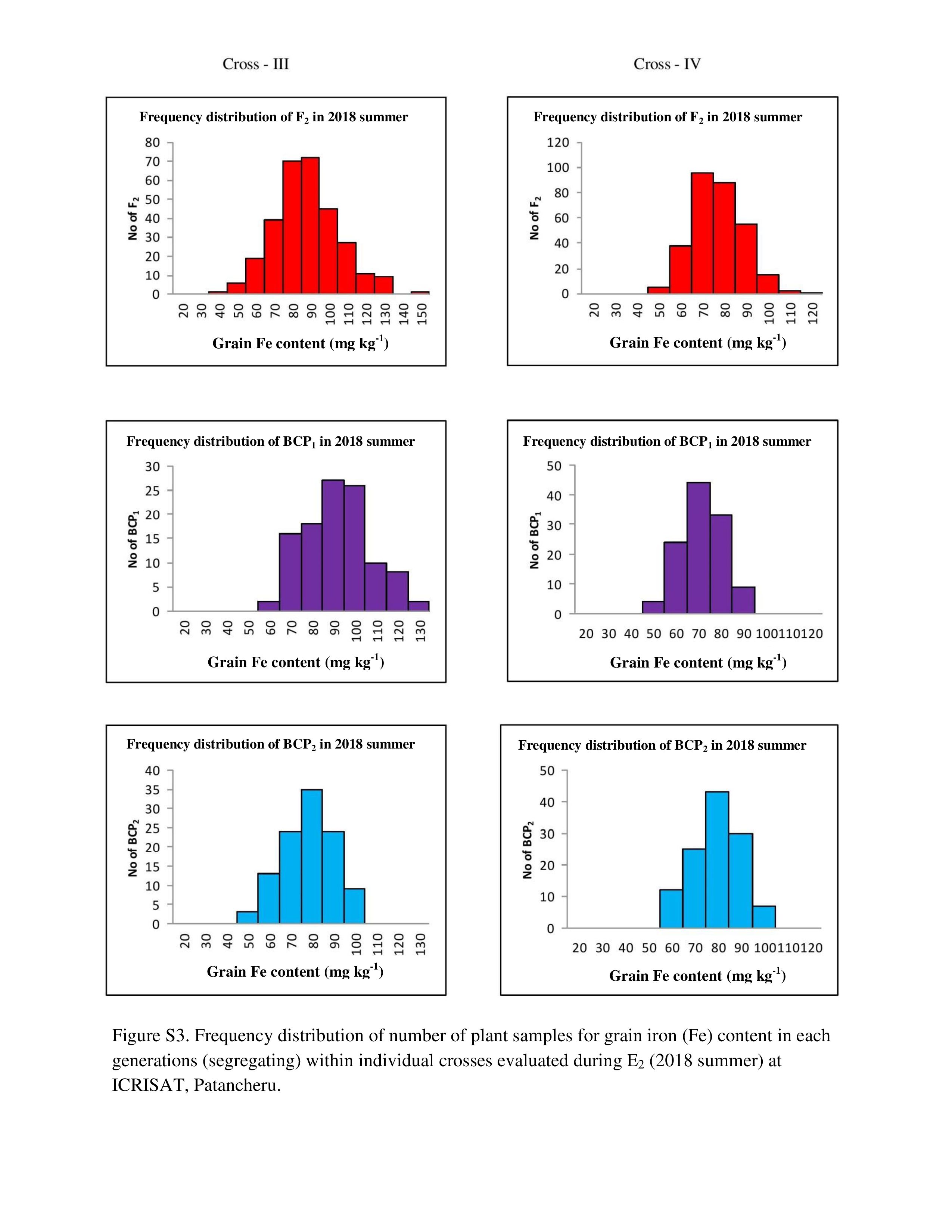

Supplement: Supplementary file 7 [file Image_6.JPEG]

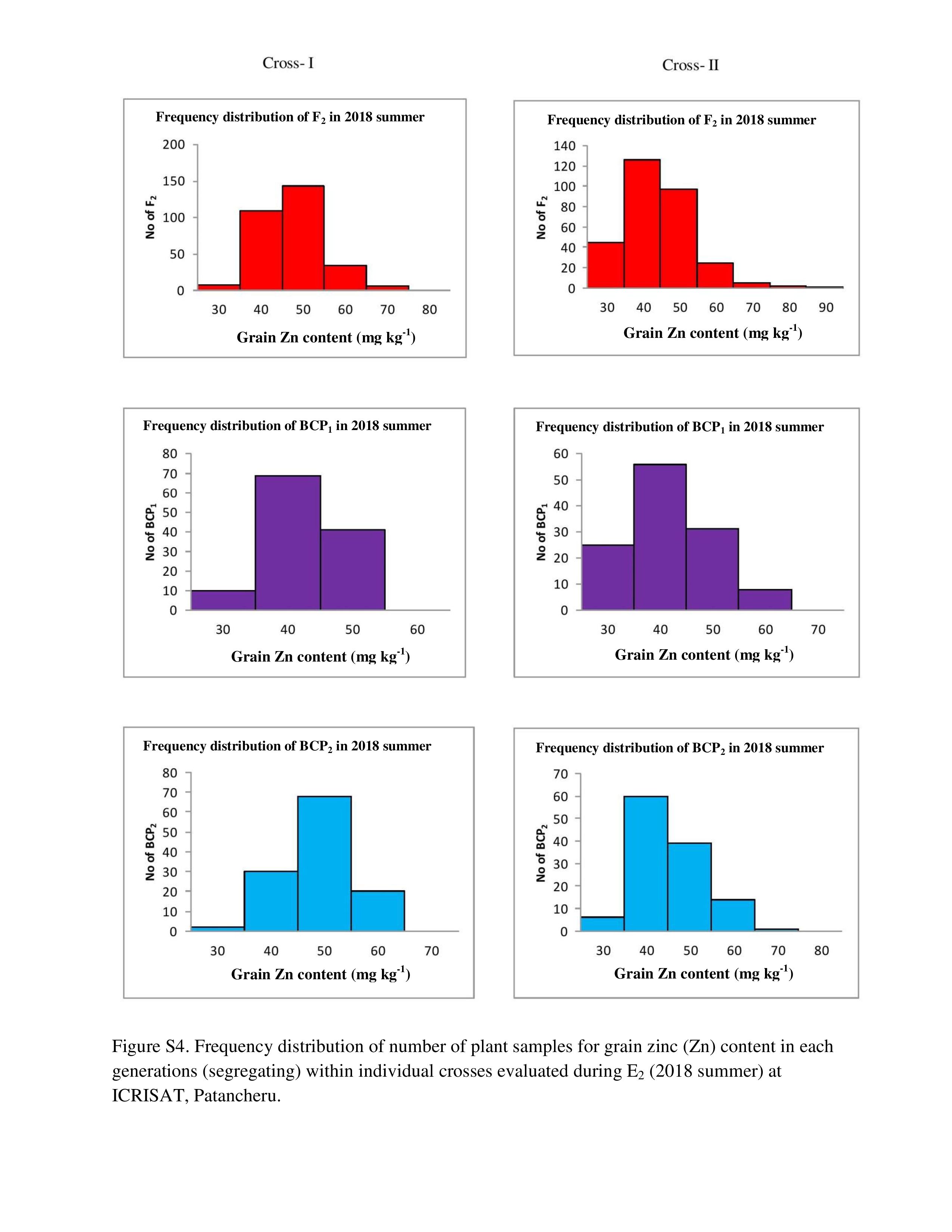

Supplement: Supplementary file 8 [file Image_7.JPEG]

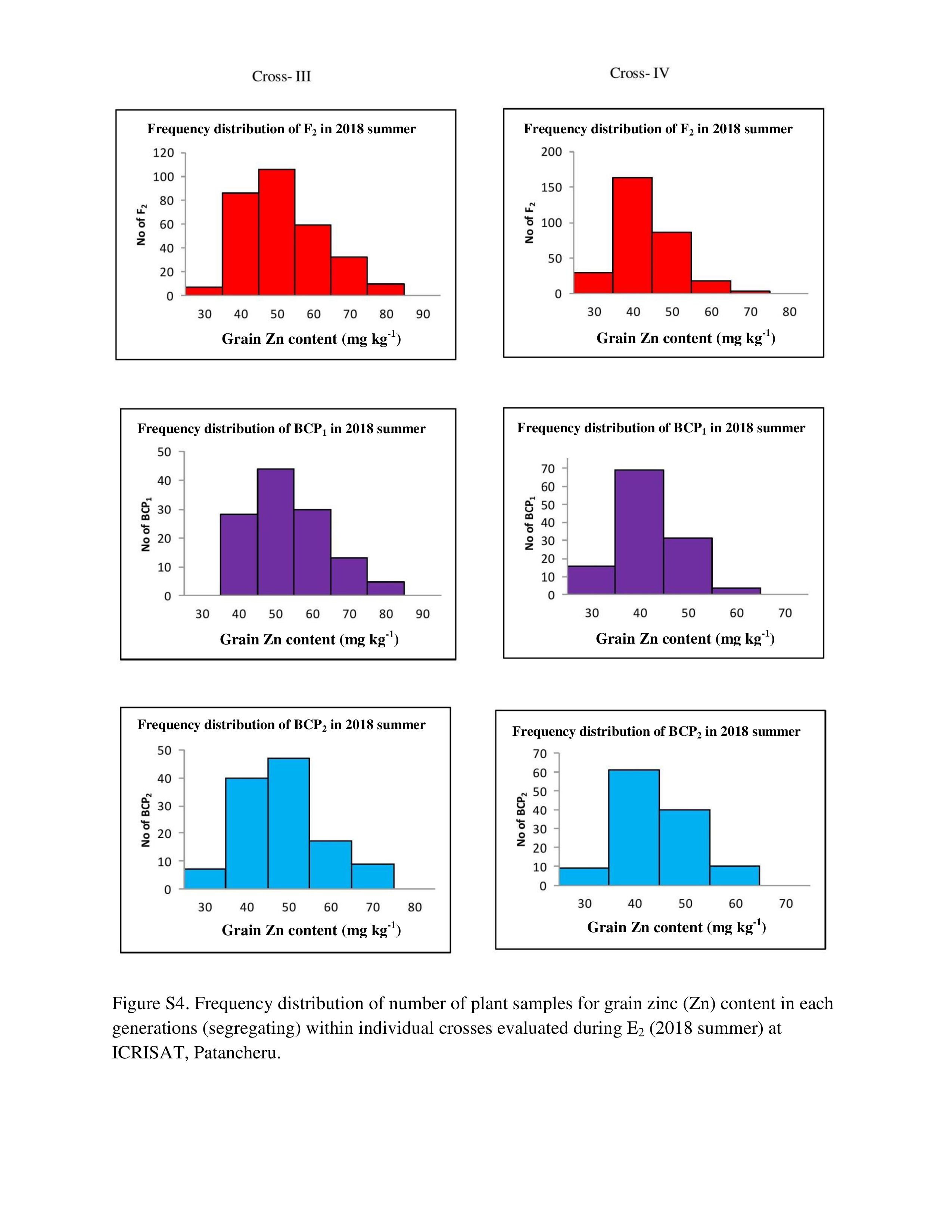

Supplement: Supplementary file 9 [file Image_8.JPEG]
